# The effect of seniority and education on departmental dictation utilization

**DOI:** 10.1186/2191-1991-1-8

**Published:** 2011-07-20

**Authors:** Kevin C Bax, Kambiz Norozi, Ajay P Sharma, Guido Filler

**Affiliations:** 1 Department of Pediatrics, Division of Gastroenterology, 800 Commissioners Road East, London, Ontario, N6A 5W9, Canada; 2 Department of Pediatrics, Division of Cardiology, 800 Commissioners Road East, London, Ontario, N6A 5W9, Canada; 3 Department of Pediatrics, Division of Nephrology, 800 Commissioners Road East, London, Ontario, N6A 5W9, Canada

**Keywords:** dictation utilization, dictation cost, training, accountability, cost effectiveness

## Abstract

**Background:**

Electronic medical records (EMR) are considered the best solution to improved dissemination of health information for patients. The associated transcription caused a significant cost increase in an academic pediatric center. An educational campaign was implemented to achieve cost-effective transcriptions without compromising the number of EMR transcriptions.

**Methods:**

We analyzed the effect of seniority on transcription times over a 4-month period. We also compared the dictation volume before and 4 months after educational interventions. This study was performed in a pediatric academic center with both inpatient and outpatient transcription utilization analyzed. All clinicians providing pediatric care and utilizing the hospital-based transcription over the study time period were analyzed. Interventions included targeted education about efficiencies in transcription, time-based dictation costs, avoidance of lengthy pauses and unnecessary detail, shortening of total transcriptions, superfluous phrases as well as structured templates. Level of training by postgraduate year of training and seniority within faculty were measured for impact on dictation time and effect of education to improve times.

**Results:**

Learners in year one had an average dictation time of 7.5 ± 2.2 minutes, which decreased with seniority to an average of 4.1 ± 2.2 minutes for senior faculty (0.0007, ANOVA). After educational initiatives were implemented, there was progressive decline in dictation utilization. The total dictation time decreased from 8,750 minutes per month in August 2009 to 4,296 minutes in December of 2009 (p = 0.0045, unpaired t-test).

**Conclusion:**

We identified a substantial need for education in dictation utilization and demonstrated that relatively simple interventions can result in substantial costs savings.

## Background

Electronic medical records (EMRs) are considered the preferred choice for the rapid dissemination of clinical information [1]. The advantages of EMR include not only dissemination of this clinical information but improved legibility and patient safety [2,3]. Our academic health science center in London, Ontario, Canada has moved towards dictations being placed within our hospital EMR. In many North American institutions, the dictation costs are absorbed within the hospital budget. As more physicians used the hospital transcription service in our center, not unsurprisingly the costs increased accordingly.

A variety of payment systems for medical transcriptions exist. After optimizing all efficiencies on the end of the transcription firm, a fixed price per minute of dictation was negotiated in our center. With increase in the usage of hospital dictation system, the transcription costs exceeded the self-imposed budget target of < 1.0% of the total hospital budget. As a cost containing measure, the hospital administration decided to invoice physicians for dictation utilization in excess of the median utilization percentage derived from peer institutions. This change in policy bore the potential of discouraging physicians to utilize the dictation system. An educational campaign was implemented to identify opportunities for succinct transcription and other cost containment strategies to maintain a similarly high proportion of transcriptions without a decrease in the usage of transcription service.

Efficiencies including voice recognition and overseas transcription had already been implemented in an attempt to hold down costs. The use of voice recognition software has not always realized cost savings particularly given the steep learning curve of most software programs to date [4]. Previously studies have identified cost of transcription as higher than anticipated [5]. The increasing usage resulted in significant cost overruns in the transcription budget for the department.

## Objective

Few reports exist about medical transcription usage in academic health science centers [6]. A study on the efficacy of strategies to contain transcription costs has been elusive. Our hypothesis was that targeted educational strategies for transcription have potential to decrease transcription costs. Additionally, we were interested in assessing if the impact of these strategies varies with the seniority level. We retrospectively analyzed the impact of educational strategies on transcription costs and any difference in the impact according to the seniority level in a single department.

## Methods

The Department of Pediatrics at the University of Western Ontario provides clinical services, both inpatient and outpatient, to a pediatric population throughout a defined geographical region of Ontario with a catchment area of 454,571 children (Provincial Council for Children's Health of the Province of Ontario, August 23, 2007). Clinical providers utilizing the transcription system include consultant physicians, advanced practice nurses, residents and clinical clerks from both London Health Science Centre and St. Joseph's Hospital in London, Ontario. The vast majority of the clinicians in the Department (74/84) utilize the hospital based transcription system.

Prior to the educational campaign and implementation of transcription streamlining methods, the transcription statistics were gathered from the transcription firm through Medical Affairs. Dictated reports for three months (June-August 2009) were analyzed. All available data from 74 clinicians (including residents) were gathered from May 2009 to August 2009 (four months). Ten staff clinicians did not utilize the central dictation system and used their secretarial support instead. Learners from other departments or institutions rotating through our program were also included. The residents were categorized according to the year of their training, ranging from year one through four. Locums and junior staff (assistant professors) were placed in one group, and all advanced practice nurses were reported in one separate group. Senior staff, associate and full professors, were placed into another group for comparison. Community pediatricians were handled separately as their dictation volumes were low and mainly related to inpatient discharge summaries. These data were compared with the time span from September to December 2009 (4 months) to evaluate the effect of the intervention.

In the fall of 2009, the Department began an educational campaign to create awareness of the current state of the department's transcription usage. The intervention consisted of written and oral communications provided to all users of the transcription system. Each individual user was also given a detail description of their transcription use compared to department averages. A variety of strategies for a reduction of overall transcription usage were identified that would not compromise the proportion of medical transcriptions on the EMR and cohesiveness of patient information. Interventions included general education about efficiencies and individual feedback. Recognizing that transcription cost was based on time, suggestions were to avoid lengthy pauses, shorten total transcriptions, avoiding unnecessary detail and superfluous phrases as well as structured templates and a focus on the current problem, medication list and impression and plan. Additional strategies included the use of templates, especially for procedures, and advanced clinical notes [1,7]. In short, advanced clinical notes reflect the semi-automated word-processing of a procedure note, summary or letter within the EMR that uses pre-existing elements and allows the cutting and pasting of laboratory data or other elements of a letter from the EMR.

We analyzed the effect of seniority on transcription times. We also compared the dictation volume before and after the educational interventions.

### Statistical analysis

Wherever possible, descriptive statistics were used. Data were then tested for normal distribution using the Shapiro Wilks test. Normally distributed data were expressed as mean and standard deviation and comparison was performed using appropriate parametric methods such as Student's t-test and ANOVA. Non-normally distributed data were expressed as median and inter-quartile range and appropriate non-parametric tests were employed. Statistical analysis was performed using GraphPad Prism version 4.01, GraphPad Software, San Diego, CA, USA.

## Results

### Pre-Intervention data

All available data from every department member that used the central dictation system were considered. Seventy-four clinicians were identified dictating between 1 and 444 clinical encounters during the initial three-month observation period. Thirty-three of these were pediatric residents (PGY1, PGY2, PGY3 and PGY4), 14 were assistant professors and 15 were associate professors or higher. In addition, 6 advanced practice nurses and six community pediatricians with more than one dictation were included.

**Figure 1:**
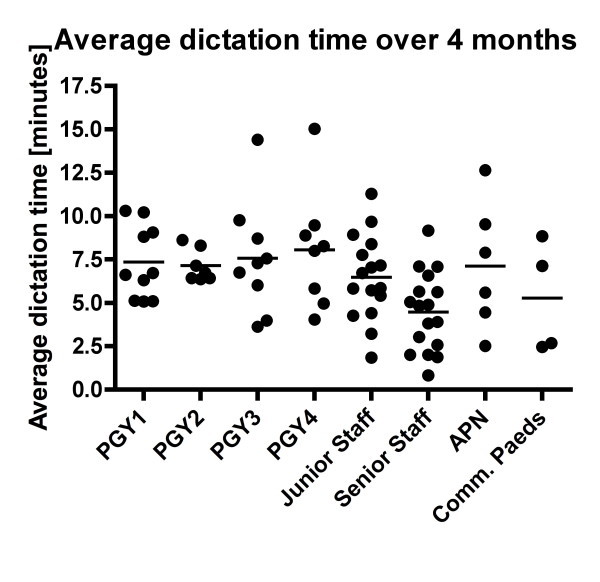
**Average dictation time [minutes/letter] per group of physicians, advanced practice nurses and community pediatricians pre-intervention**. There was a significant decrease of the average dictation time per transcript with the increase in seniority. Learners in year one had an average dictation time of 7.5 ± 2.2 minutes which decreased with seniority to an average of 4.1 ± 2.2 minutes for senior faculty (0.0007, ANOVA).

The median number of dictations per individual was 12.25 per month (2.75-25^th ^percentile, 26.13 - 75^th ^percentile, range 0.25 to 111 dictation per month). The median total dictation time per month per person was 66.71 minutes (19.24 min - 25^th ^percentile, 149.6 min - 75^th ^percentile, range 0.67 to 360.8 minutes/month). The median length of dictation was 6.5 minutes/letter (4.65 min - 25^th ^percentile, 8.29 min - 75^th ^percentile, range 0.82 to 15.03 minutes/letter). Table 1, 2 and 3 and Figure 1 outline the breakdown of dictation number, time, and average time per dictation categorized according to the level of training over the four months pre-intervention.

**Table 1:** Median number of dictations per group of physicians, advanced practice nurses (APN) and community pediatricians over the 4 month study period

	Level of Training							
	
Characteristic	PGY1	PGY2	PGY3	PGY4	Junior	Senior	APN	Community
Number	11	7	6	9	14	15	6	6

25% Percentile	6	27	3	3.	11	64	8.5	1

Median	27	66	31.5	24	129	106	52.5	16.5

75% Percentile	46	90	75	60	176	143	119.5	44

PGY1-4 = Post Graduate Year One to Four, Junior = Junior Faculty, Senior = Senior Faculty, Community = Community Pediatrician

**Table 2:** Median total dictation time per month in minutes per group of physicians, advanced practice nurses (APN) and community pediatricians over the 4 month study period

	Level of Training							
	
Characteristic	PGY1	PGY2	PGY3	PGY4	Junior	Senior	APN	Community
Number	11	7	6	9	14	15	6	6

25% Percentile	61.84	162.5	29.57	23.93	7.660	190.4	10.52	5.76

Median	233.8	547.8	177.8	213.4	163.1	506.2	87.96	48.62

75% Percentile	281.2	621.0	519.3	387.4	697.0	729.5	565.7	251.9

**Table 3:** Mean dictation time per transcript [min], stratified by group of physicians, advanced practice nurses and community pediatricians

	Level of Training							
	
Characteristic	PGY1	PGY2	PGY3	PGY4	Junior	Senior	APN	Community
Number	11	7	6	9	14	15	6	6

Mean	7.316	7.030	7.605	7.906	6.618	4.143	7.107	4.838

Std. Deviation	1.938	1.011	2.199	3.316	2.183	2.212	3.679	2.641

Std. Error	0.584	0.382	0.8976	1.105	0.583	0.571	1.502	1.078

From PGY1 to Senior staff, dictation times decreased significantly (p = 0.0007, ANOVA). Average time per dictation decreased significantly between junior and senior staff (p = 0.0053, unpaired t-test).

Among different groups, there was a significant decrease in the average dictation time per transcript with the increase in seniority. Learners in year one had an average dictation time of 7.4 minutes/letter, which decreased with seniority to an average of 4.5 minutes/letter for senior faculty (p = 0.0075, ANOVA, Table 3, Figure 1). Among the pediatric residents, transcript time per dictation was higher in year 3 and 4 residents as compared with year 1 and 2 residents. This could be due to the rotation schedule as the residents in year two mostly go through their subspecialty rotations, and years three and four involve their in-patient rotations necessitating longer admission notes and discharge summaries.

We were able to calculate the differences by gender. Forty-five of the physicians were female (61%). Mean dictation time for female physicians was 7.1 ± 2.3 min, significantly longer than the 5.3 ± 2.9 min for the males (Student's t-test). The number of dictations per month was not significantly different when analyzed by gender, but the total dictation length per month was longer (females median 69.8 minutes/month versus males median 47.6 minutes/month, although this did not reach statistical significance, p = 0.3032, Mann Whitney test). It is noteworthy that only 6 of 33 the residents were male, which confounded these results.

### Post-Intervention data

**Figure 2:**
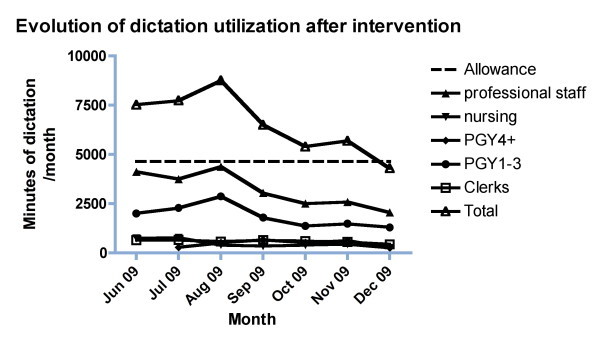
**Evolution of dictation utilization 3 months before and 4 months after intervention**. The months June to August 2009 reflect the time period before the intervention and the time period from September 2009 to December 2009 reflects the intervention period. There was a significant decrease of dictation time from 8,750 minutes per month in August 2009 to 4,296 minutes in December of 2009. When comparing four months before and four months after intervention, the dictation times were significantly different (p = 0.0045, unpaired t-test).

Following the interventions outlined above, there was a progressive decline of the dictation utilization. The total dictation time for all physicians decreased from 8,750 minutes per month in August 2009 to 4,296 minutes in December of 2009 (Figure 2). Details of the total dictation times per month are provided in Table 4. Importantly, there was a slight drop from an average 1,315 dictations per month to 921 dictations per month (minus 30%). This drop could be partly explained by higher usage of alternative transcription strategies such as advanced clinical notes. Currently, our system does not allow us to track advanced clinical notes. Some of the dictations were transcribed by medical secretaries of the staff and uploaded on the EPR outside of the central dictation system. Others were also transcribed as advanced clinical notes, which allow the physicians the updating of a previous transcription note on the EMR for the recent clinical encounter. The current system does not capture the usage of these alternative transcription strategies. Importantly, the average time per dictation decreased from 5.38 minutes/dictation (pre-intervention period) to 3.75 minutes/dictation (post-intervention period) (p = 0.0014, Wilcoxon's matched pairs test). A significant pronounced effect was found with faculty members (reduction from 5.19 ± 2.65 minutes/dictation to 3.77 ± 1.98, p = 0.0037, paired t-test).

**Table 4:** Dictation utilization time (minutes) before (June to August 2009) and after intervention.

Month	Allowance	Professional staff	nursing	PGY4+	PGY1-3	Clerks	Total
May 2009	4648.4	3627.0	711.3	238.12	1181.3	N/A	5757.7

June 2009	4648.4	4122.6	749.6	0.0	2005.5	646.3	7524.0

July 2009	4648.4	3745.7	767.1	275.2	2287.0	662.0	7736.9

August 2009	4648.4	4379.4	402.7	523.4	2868.5	576.4	8750.4

September 2009	4648.4	3047.9	346.8	667.1	1795.6	643.7	6501.1

October 2009	4648.4	2504.0	398.6	517.6	1367.8	603.9	5391.8

November 2009	4648.4	2586.8	442.2	628.6	1488.2	554.0	5699.8

December 2009	4648.4	2057.1	253.4	252.9	1299.9	433.3	4296.7

Allowance means the dictation time that the department was permitted to use.

## Discussion

The objective of this manuscript was to describe whether there was an association between seniority and dictation lengths and the effect of education on dictation utilization in a clinical department at an academic health science center. A literature search revealed limited data. Two important observations were made: Learners had the longest dictation times, and senior staff had shorter dictation times per transcript than junior staff. Secondly, we observed a significant reduction of total transcription times and dictation time per transcript after the intervention. Apart from a reduction of the dictation time per transcript, there was also a decrease of the average number of transcripts per month. The proportion of alternative transcripts is unknown. Transcripts were a mix of discharge summaries and outpatient notes. We currently do not have a system that allows us to track the type of dictation separately. It is possible that residents dictated more discharge summaries than staff. Discharge summaries tend to be longer. This may have confounded the results. However, a significant numbers of staff who dictated large volumes were inpatient attendings. There is also some mild seasonal variability with reduced outpatient volumes in the early August and in the last week of December. Both periods were included in the analysis, therefore seasonal variability should not have affected the results.

There is limited information about the dictation efficiency of physicians. Lawler [5] noted that more senior faculty has more efficient transcription utilization. Our data confirm these findings and demonstrate a progressive shortening of dictation time per transcription with seniority. This is not surprising. It is important that learners be given adequate education on medical transcription. There is abundant data that demonstrates that learners and junior faculty exhibit less efficient utilization of health care dollars. As pointed out by Lawler [5], seniority was inversely correlated with transcription costs, and "specific education regarding dictation form and content and ways to decrease these costs is appropriate". The authors are concerned that insufficient emphasis is placed on this education component in medical school and residency programs.

While in most North American academic centers the responsibility of medical records lies with the hospitals, accountability and fiscal responsibility should lie with all medical staff. Traditionally, the cost for medical transcription has not been shared with the staff. When canvassing the staff, nobody was aware of the costs. The substantial increase of the transcription costs forced the hospital administration to address the issue and it was reasonable to call for some accountability from the physicians. There was concern that defraying the costs to the physicians might result in reduced transcription and a lower proportion of transcripts on the EMR.

Our manuscript clearly demonstrates that education can reduce costs. It is to be acknowledged that the data collection cannot provide for a complete assessment of the proportion of records placed on the EMR. One of the strategies involved use of advanced clinical notes and templates. In another study, the utilization of templates has proven to improve the quality of the EMR [8]. We also cannot comment whether completeness of documentation was lost with the intervention or whether there was a negative effect on patient safety.

It is important to highlight that these results are from a relatively small tertiary care Children's Hospital with many subspecialists and only 74 clinicians, and it remains questionable whether these results can be generalized to a more homogenous group of physicians. The effect of seniority should be assessed by other groups in a larger sample size. The total number of 5,125 dictations, however, is of sufficient sample size. Nonetheless, this study clearly demonstrates that education about medical transcription costs and efficiencies can result in substantial savings. Our study suggests that focusing on junior faculty and learners should be a priority. Further research is required to assess that the proportion of transcripts on the EMR and their quality remains unaffected. Finally, better data on patient safety in relationship to completeness of EMR records is required.

## Conclusions

Transcriptions costs comprise an important and unappreciated part of the budget of a health care institution. Accountability and fiscal responsibility of the physicians should be mandated. We identified a substantial need for education in this field and demonstrated that relatively simple intervention can result in substantial costs savings.

## Competing interests

The authors declare that they have no competing interests.

## Authors' contributions

KB helped in the design of this study and drafted the manuscript. KN participated in helping review the statistical analysis and the critical review of the manuscript. AS participated in the critical review of the manuscript and offered suggestions for the design of the study. GF participated in the design and conception of the study, arranged for the statistical analysis and extensively edited the manuscript. All authors read and approved the final manuscript.
